# Assessing Community Readiness to Reduce Childhood Diarrheal Disease and Improve Food Security in Dioro, Mali

**DOI:** 10.3390/ijerph13060571

**Published:** 2016-06-08

**Authors:** Erica C. Borresen, Cordelia Stone, Abdoulaye Boré, Alima Cissoko, Ababacar Maiga, Ousmane A. Koita, Elizabeth P. Ryan

**Affiliations:** 1 Department of Environmental and Radiological Health Sciences, Colorado State University, Fort Collins, CO 80523, USA; erica.borresen@colostate.edu (E.C.B.); cordstone@gmail.com (C.S.); 2 Faculté des Sciences Humaines et des Sciences de l’Education, University of Letters and Human Sciences of Bamako, Bamako, BP 2191, Mali; abdoubore@yahoo.fr; 3 Laboratoire of Biologie Moléculaire Appliquée, University of the Sciences, Techniques and Technologies of Bamako, Bamako, BP 1805, Mali; alimacissoko@yahoo.fr (A.C.); maigababacar@yahoo.fr (A.M.)

**Keywords:** community readiness, childhood diarrhea, food security, community interventions, Mali

## Abstract

Diarrhea and malnutrition represent leading causes of death for children in Mali. Understanding a community’s needs and ideas are critical to ensure the success of prevention and treatment interventions for diarrheal disease, as well as to improve food security to help reduce malnutrition. The objective of this study was to incorporate the Community Readiness Model (CRM) for the issues of childhood diarrheal disease and food security in Mali to measure baseline community readiness prior to any program implementation. Thirteen key respondents residing in Dioro, Mali were selected based on varied social roles and demographics and completed two questionnaires on these public health issues. The overall readiness score to reduce childhood diarrheal disease was 5.75 ± 1.0 standard deviation (preparation stage). The overall readiness score to improve food security was 5.5 ± 0.5 standard deviation (preparation stage). The preparation stage indicates that at least some of the community have basic knowledge regarding these issues, and want to act locally to reduce childhood diarrhea and improve food security and nutrition. Proposed activities to increase community readiness on these issues are provided and are broad enough to allow opportunities to implement community- and culturally-specific activities by the Dioro community.

## 1. Introduction

Mali, located in the Sahel of West Africa, covers approximately 475,000 square miles and has a population of 15 million. It shares borders with seven countries. Current country health statistics indicate that 38% of children under five are stunted, 13% are wasted, and 82% are anemic [1]. The long-term effects of food insecurity, particularly in children, lead to malnutrition, stunting, and impaired cognition [2]. These outcomes share complex relationships with diarrheal diseases, and may involve a subclinical gut inflammation and altered gastrointestinal structure, referred to as environmental enteric dysfunction (EED) [3]. These global public health issues merit sustainable solutions that are acceptable to the community and require engaged collaborations from multiple stakeholders across biomedical, agriculture, nutrition, public health, toxicology, engineering, and maternal and child health sectors [4]. Furthermore, a World Bank study estimated that Mali loses over $235 million in gross domestic product annually to vitamin and mineral deficiencies warranting immediate attention with sustainable solutions [5]. Current community perceptions and behaviors to address diarrheal disease and food security are essential to consider prior to any program implementation. Health behavior change at the community level and identification of existing community resources and national nutrition program policies will incite co-creation of programs that are socio-culturally motivated and engage positive health promotion behaviors [6]. The Community Readiness Model (CRM) is a tool that integrates social and cultural contexts to understand how an issue is perceived by the community and what types of strategies can be developed at the community level. The model was developed at the Tri-Ethnic Center for Prevention Research at Colorado State University (Fort Collins, CO, USA) to assess the level of readiness to engage in prevention activities targeting a particular issue [7]. A systematic review of CRM from the scientific literature demonstrated applications including, but not limited to facilitating community-based change, creating interventions that are community- and culturally-specific, and building cooperation among systems and individuals [8]. 

The objective of this study was to determine baseline community readiness scores for Dioro (a rice-producing community in Mali) on the public health issues of childhood diarrheal disease and food security, as both of these community issues are related and integrated with regards to child health. It is important to note that while this CRM assessment focused on the issue of food security as a means to reduce malnutrition, metrics for nutrition security are emerging to address the complexity of malnutrition, such that nutritious food is not only available and accessible, but also appropriately utilized by everyone, especially children [9]. Findings from the community readiness analysis will aid our translational research and development team to co-create an environment for participatory-driven strategies that promote healthy behaviors that are aimed to prevent or treat diarrheal disease in children, as well as to improve food security throughout the community.

## 2. Materials and Methods 

### 2.1. The Community Readiness Model (CRM)

**Figure 1:**
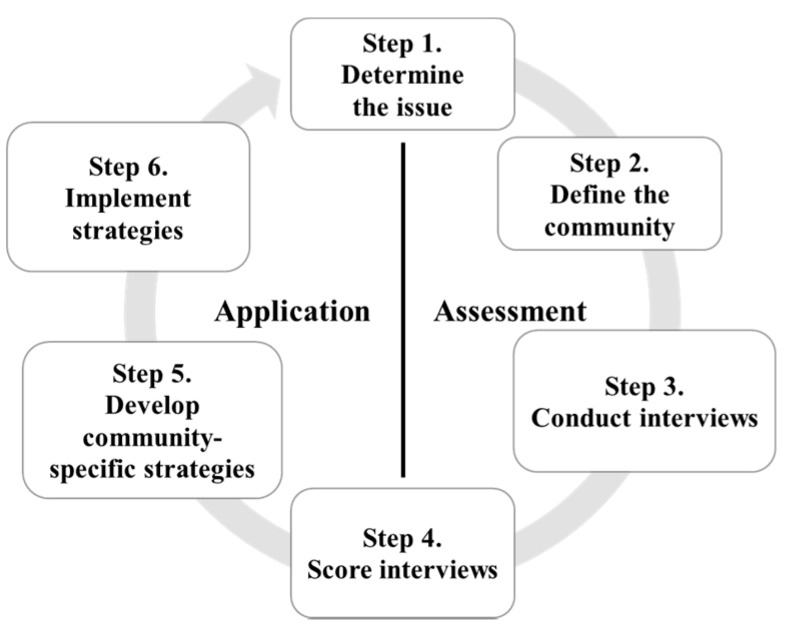
Step-by step process of community readiness. The first phase of community readiness (assessment) includes steps 1–4. Step 4 (scoring the interviews) determines the readiness level (1–9) of a community. The second phase (application) includes steps 5–6 and involves the community to develop and implement strategies based on the stage of readiness.

Figure 1 provides an overview of the 6-step CRM assessment and application process. The assessment phase has four steps: (1) identify the issue; (2) define the community with respect to the issue; (3) conduct interviews with key respondents; and (4) score the interview responses. The second phase of community readiness is the application phase, and it has the final steps: (5) develop community-specific strategies based on the stage of readiness as determined in the assessment phase and (6) implement those strategies.

**Figure 2:**
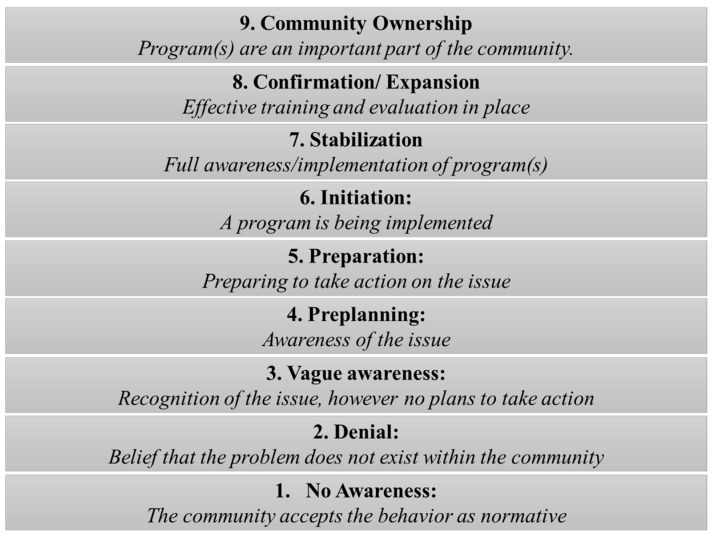
Nine stages of community readiness, as determined through the scoring process.

To conceptualize a community’s readiness, nine stages are defined as part of the CRM and include: (1) No Awareness: the community accepts the behavior as normative; (2) Denial: belief that the problem does not exist within the community; (3) Vague Awareness: recognition of the issue, however no plans to take action; (4–5) Awareness of the issue and Preparing to take action; (6) Initiation: indicates a program is being implemented; and (7–9) Full awareness/implementation of programs, including effective training and evaluation that leads to a high level of community ownership (Figure 2). It is the scoring of the interview responses (Step 4) that determines which of the nine stages best describes the readiness level of the community. These stages are similar to the transtheoretical model with a focus on community behavior change rather than individual behavior change [7].

This article provides results on the first four steps as an appropriate baseline before an intervention is developed in the community, as well as indicates proposed activities to begin the 5th step of developing community-specific strategies [8].

### 2.2. Community, Study Population, and Interview Process

Thirteen key respondents living and working in Dioro, Mali (located 275 km from the capital of Bamako) were selected to be interviewed for the CRM assessment using two questionnaires developed to assess childhood diarrheal disease and food security as they pertain to the Dioro community. These individuals were chosen based on their social role within the community—particularly when it came to knowledge about childhood health, agricultural production, involvement in community and government organizations, as well as representing Dioro’s eight districts. The questions were developed to determine the level of readiness for each of the following dimensions: (1) Knowledge of efforts; (2) Leadership; (3) Community climate; (4) Knowledge of issue; and (5) Resources. Additional questions pertaining to health and food behaviors of the Dioro community were included, but were not part of the CRM assessment. The responses to these additional questions provide important community information to be used during the development and implementation of community-specific strategies (Application Phase). The protocol for the study and the questionnaires (written in English first, and then translated to French and Bambara languages) were reviewed and approved by the Colorado State University Research Integrity and Compliance Review Office in Fort Collins, CO USA and the Institut National de Recherche en Santé Publique in Bamako, Mali (protocol #15-5727H). All interviews were conducted between June and July 2015 and questions were asked via an audio recording of a well-known individual in the village to maintain consistency in how the questions were provided and to ensure the questions were asked in the correct Bambara dialect from Dioro. Verbal consent was obtained prior to conducting the interviews and participants were reimbursed $2 (USD) for their time. Key respondent interviews were completed at a central facility at the Dioro clinic and were audio recorded. These recordings were translated from French or Bambara into English for scoring purposes.

### 2.3. CRM Scores and Analysis

Each completed interview received five readiness scores, one for each dimension, using dimension-specific rating scales. The scores were then averaged by dimension across all key respondents to provide a profile of dimension readiness scores for the Dioro community. Each dimension score corresponded to a stage of readiness, along with an overall average readiness score that was calculated from the five dimension scores. All scores were confirmed between two trained CRM scorers and there were no major deviations to the scoring protocol. The two questionnaires are included as supplementary data (Supplementary Materials). The additional health and food behavior questions used in the interview (but not part of the readiness scoring) are also included.

## 3. Results

### 3.1. Participant Characteristics

Thirteen key respondents completed two CRM questionnaires on the issues of childhood diarrhea and food security. Four respondents were female (31%) and nine were male (69%). All lived in various neighborhoods of Dioro and ranged in age from 25 to 65 years old. These individuals were chosen to be key respondents based on their various areas of expertise. They ranged from a well-known radio spokesperson to a teacher to a health worker to a rice farmer. Table 1 illustrates the key respondent characteristics. Although thirteen key respondents were interviewed, a few interviews were not included in the final scoring due to lack of appropriate answers to the questions and the inability to provide a proper score. Ten respondents were used in the childhood diarrheal disease scoring and twelve respondents were used in the food security scoring.

### 3.2. Community Readiness on Childhood Diarrheal Disease

**Figure 3:**
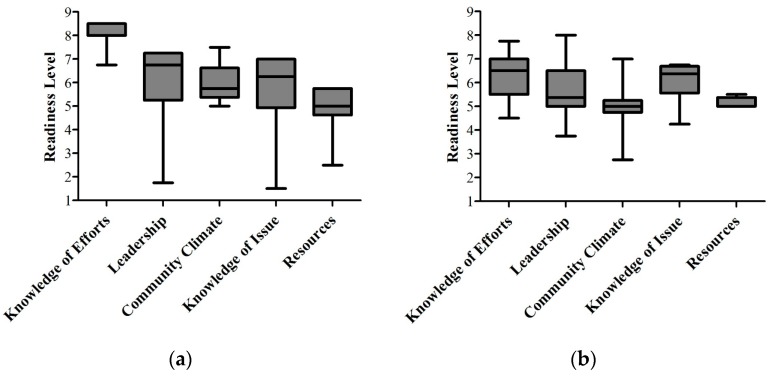
Community readiness results for (**a**) reducing childhood diarrheal diseases and (**b**) improving food security that show the full range of scores across each dimension (Median plus minimum and maximum scores).

The community readiness results assessing childhood diarrheal disease are provided in Table 2. The overall readiness score was 5.75 ± 1.0 standard deviation (SD), indicating that the community readiness level is at the preparation stage. In regards to specific readiness dimensions, Knowledge of Efforts received a score of 8.0 ± 0.5 SD, the confirmation stage. Leadership scored a 5.75 ± 1.75 SD, which falls into the preparation stage. Community Climate had a score of 5.75 ± 0.75 SD, again in the preparation stage. Knowledge of Issue also was in the preparation stage with a score of 5.5 ± 1.75 SD. Resources received a score of 4.75 ± 1.0 SD, the preplanning phase. Figure 3a illustrates the range of scores across each dimension based on all key respondents’ answers.

### 3.3. Community Readiness on Food Security

The community readiness results assessing food security are provided in Table 2. The overall readiness score was 5.5 ± 0.5 SD, which is the preparation stage of readiness. For specific dimensions, Knowledge of Efforts scored a 6.25 ± 1.0 SD, the initiation stage. Leadership, Community Climate, Knowledge of Issue, and Resources all scored within the preparation stage with scores of 5.5 ± 1.0 SD, 5.0 ± 1.25 SD, 5.75 ± 1.0 SD, and 5.0 ± 0.5 SD, respectively. Figure 3b illustrates the range of scores across each dimension based on all key respondents’ answers.

### 3.4. Collection of Health and Food Behaviors within the Community

In addition to asking questions based on the CRM, the interviewers were allowed to collect information to help develop community-driven strategies for implementing specific activities focused on both reducing childhood diarrheal diseases and improving food security. When asked what types of community leaders would need to be involved in these efforts, key respondents stated that the village chief, imams, community health workers, the Health Center Board, women and youth groups, the Coordination of Associations and NGOs of Women in Mali (CAFO), farmers, and rice millers should participate and be engaged.

In regards to current efforts occurring to improve these issues, key respondents provided information about programs that the community is familiar with and participates in, as resources are available. These efforts include educational programs to wash hands and clothes with soap and hygiene programs that include water, sanitation, and hygiene (WaSH). The local health center and its network of community health workers distribute zinc and oral rehydration salt solutions, as well as ready-to-use therapeutic foods for severe and moderately acute malnourished children, and monitor breastfeeding.

Additionally, an open-ended question was asked to get an idea on the major indications that influence reducing childhood diarrheal disease in Dioro. While WaSH strategies were mentioned, the key respondents also noted that safe and nutritious food was important, further indicative of an existing understanding of the complex connections between food systems and health.

### 3.5. Initial Proposed Activities to Improve Readiness

The CRM provides proposed activities to increase readiness to the next stage that are intended to keep the community on track for addressing the issue in a more culturally acceptable way. Table 3 and Table 4 include a descriptive interpretation of the scores for each dimension, based on the readiness scores received from this study, as well as recommended activities to move the community to a higher stage of readiness. These proposed activities are broad enough to allow the community and invested groups of stakeholders the opportunity to determine and implement specific activities.

## 4. Discussion

The main purpose of this study was to conduct two CRM assessments in a rice growing community in Mali geared towards identifying strategies to address the issues of childhood diarrheal disease and food security to allow for future community-based projects. These baseline community readiness scores for the community of Dioro can be used as a starting point to organize activities that are specific to the community needs and ideas.

For both the issues of reducing childhood diarrheal diseases and improving food security, the community’s overall readiness scores indicate that at least some members in the community have basic knowledge regarding these issues, are concerned, and want to do something locally about childhood diarrhea and food security. The assessments helped to collect important information on what community leaders and organizations should be involved in the planning, what resources are currently available that address these issues, and what can influence these issues the strongest. The purpose of using key respondents was to collect information from a wide range of people in the community, including community leaders, professionals in the field, and residents, all of whom have the knowledge and experience in the community as it relates to the issue being assessed.

Most of the community members of Dioro have a clear recognition that childhood diarrheal diseases and food security are a local problem, but given the absence of focused or detailed efforts (besides going to the health center for treatment), we confirmed that limited prevention efforts are in place. Results from the CRM assessment indicate that substantial additional initiatives are needed to expand benefits on a community level from these programs and include the degree of success to reduce childhood diarrheal diseases and food security. Given the breadth of community knowledge that our key respondents provided, these baseline scores should be considered representative of the current readiness of Dioro residents on these important issues.

The additional health and food behavior questions found that rice is a very important staple food in this community as it is easy to access since it is grown there and is part of the main meal. Key respondents noted that there was interest in the community to modify ingredients of traditional foods as long as it elevated taste, was convenient, was nutritious, able to improve health, and that the community is well-informed of what the modifications are. Having an overall score of 5.5 (Preparation) for community readiness in regards to improving food security, Dioro represents a promising community for implementation of novel food-focused projects. This community is aware of food security issues, but requires practical, concerted efforts to be put in place to address and improve the current situation, such as proper storage units to store food to be used during times of limited food access.

This study had limitations and important lessons learned when applying the CRM assessment to an international setting. One limitation was that the CRM assessment was developed in English and then translated into French and Bambara. The responses were also recorded in French and Bambara and translated into English for scoring and analysis. While some information may be lost in translation, our research group standardized the minimum number of translators based on fluency in all three languages. Also, while the CRM requires 4–6 key respondents within a community, this study utilized 10 key respondents for the issue of childhood diarrheal disease and 12 for the issue of food security. The additional number of individuals helped strengthen the responses to each question since each interview had to be translated for scoring purposes. This group of key respondents was a good representation of the Dioro community that incorporated a breadth of social roles and professions, especially by including women. Additionally, the questionnaire assessing food security was developed and translated prior to the availability of the expanded version of the CRM questionnaire. Since each key respondent had to complete two questionnaires in one sitting, it was decided to keep the food security questionnaire in its simplified form to reduce interviewer/interviewee fatigue. The different versions were scored following the same CRM scoring protocol.

Finally, Dioro was part of the Millennium Villages Project, an international nonprofit organization focused on accelerating the Millennium Development Goals that ended in 2015 [11]. This designation allowed for specific development initiatives (e.g., road construction, electricity, drinking water) to advance in this region of Mali, particularly infrastructure and accessibility to nutritional supplementations to treat severely and moderately acute malnourished children. Participation in the Millennium Villages Project is a major strength of the Dioro community’s awareness to local public health issues and efforts, including childhood diarrheal disease, malnutrition, and food security, which may explain the high readiness scores, especially in the knowledge of efforts dimension.

## 5. Conclusions

Issues of childhood diarrheal diseases and food security have not previously been assessed through the CRM, and particularly in a rural, developing country setting. Due to the complexity of these global health issues and the high level of urgency to address childhood diarrhea and malnutrition in Mali, it is undoubtedly essential to integrate the community viewpoints and attitudes in these discussions and to verify how invested they are in resolving these issues [6].

This study provided baseline community readiness scores for a rice-producing community of Dioro in Mali on public health issues of childhood diarrheal diseases and food security. Next steps in this initiative will be for the Dioro community to develop and implement sustainable, community- and culturally-specific strategies based on these baseline readiness stages. In addition to a focus on reducing childhood diarrheal diseases and improving food security, future plans should also include a reassessment of readiness after community interventions take place. Moreover, continuous monitoring and evaluation of whether these public health issues begin to resolve locally will be an important outcome measure for the community.

## Figures and Tables

**Table 1:** Key respondent characteristics (*n* = 13).

Characteristic	Number of Key Respondents (%)
Sex	
Male	9 (69%)
Female	4 (31%)
Age range	
25–34 y	2 (15%)
35–44 y	2 (15%)
45–54 y	1 (10%)
55–64 y	4 (30%)
65+ y	4 (30%)
Dioro neighborhood	
Bozola	3 (23%)
Darsalam	2 (15%)
Djeda	1 (8%)
Hamdalaye	2 (15%)
Mairie	2 (15%)
Médine	1 (8%)
Missira	1 (8%)
Tinti	1 (8%)
Social roles/Professions	
CAFO ^1^	2 (15%)
Community Health Worker	1 (8%)
Fisherman	1 (8%)
Merchant	2 (15%)
Neighborhood chief	2 (15%)
Radio director	1 (8%)
Rice farmer	2 (15%)
Teacher	2 (15%)

**^1^** CAFO = Coordination of Associations and NGOs of Women in Mali.

**Table 2:** Community readiness scores following analysis of individual interviews, were averaged for each dimension and the overall readiness score for reducing childhood diarrheal diseases and improving food security **^1^**.

Community Issue	Knowledge of Efforts	Leadership	Community Climate	Knowledge of Issue	Resources	Overall Readiness
Childhood diarrheal disease	8.0 ± 0.5 (Confirmation)	5.75 ± 1.75 (Preparation)	5.75 ± 0.75 (Preparation)	5.5 ± 1.75 (Preparation)	4.75 ± 1.0 (Preplanning)	5.75 ± 1.0 (Preparation)
Food security	6.25 ± 1.0 (Initiation)	5.5 ± 1.0 (Preparation)	5.0 ± 1.25 (Preparation)	5.75 ± 1.0 (Preparation)	5.0 ± 0.5 (Preparation)	5.5 ± 0.5 (Preparation)

**^1^** Values are reported as: Average Readiness Level ± Standard Deviation (Readiness Level).

**Table 3:** Proposed activities to increase community readiness for reducing childhood diarrheal diseases according to each dimension (adapted from [10]).

Dimension	Score/Stage	Stage Definition	Goal to Maintain/Improve Stage	Activities to Increase Stage
Knowledge of Efforts	8.0/Confirmation	Most community members know a great deal about local efforts.	Conduct training and meetings to review progress and modify programs	Develop formal network Prepare community specific risk profile
Leadership	5.75/Preparation	Leadership is participating in developing and improving efforts. Some leaders are playing a key role.	Gather existing information to help plan strategies	Utilize community leaders to speak to groups and participate in local radio talk shows
Community Climate	5.75/Preparation	The community’s attitude is concerned about this issue and wants to do something about it.	Gather existing information to help plan strategies	Conduct community surveys and public forums to develop strategies Sponsor a community event to initiate the effort
Knowledge of Issue	5.5/Preparation	The community members have basic knowledge about causes, consequences, signs and symptoms of childhood diarrheal diseases.	Gather existing information to help plan strategies	Conduct community surveys Present in-depth local information
Resources	4.75/Preplanning	There are limited resources currently available to address the issue.	Raise awareness with concrete ideas	Review existing efforts in community to determine who benefits and what the degree of success has been

**Table 4:** Proposed activities to increase community readiness for improving food security according to each dimension (adapted from [10]).

Dimension	Score/Stage	Stage Definition	Goal to Maintain/Improve Stage	Activities to Increase Stage
Knowledge of Efforts	6.25/Initiation	Most community members have at least basic knowledge of local efforts.	Provide community specific information	Plan publicity efforts associated with start-up of program/activity
Leadership	5.50/Preparation	Leadership is participating in developing and improving efforts. Some leaders are playing a key role.	Gather existing information to help plan strategies	Utilize key leaders and influential people to speak to groups and to participate in local radio shows
Community Climate	5.0/Preparation	The community’s attitude is concerned about this issue and wants to do something about it.	Gather existing information to help plan strategies	Conduct community surveys and public forums to develop strategies Sponsor a community event to initiate the effort
Knowledge of Issue	5.75/Preparation	The community members have basic knowledge about causes and consequences regarding lack of food security.	Gather existing information to help plan strategies	Conduct community surveys Present in-depth local information.
Resources	5.0/Preparation	There are some resources identified that could be used to address the issue.	Gather existing information to help plan strategies	Conduct formal surveys with stakeholders to determine long-term plan of resources (both monetary and non-monetary)

## Supplementary Materials

The following are available online at www.mdpi.com/1660-4601/13/6/571/s1, Assessing community readiness to reduce childhood diarrheal disease questionnaire, Assessing community readiness to improve food security questionnaire.
