# Supplementary material for: Assessing Community Readiness to Reduce Childhood Diarrheal Disease and Improve Food Security in Dioro, Mali
Source: Int J Environ Res Public Health. 2016 Jun 8;13(6):571. doi: 10.3390/ijerph13060571 (PMC4924028; doi:10.3390/ijerph13060571)
Supplement: Supplementary file 1 [file ijerph-13-00571-s001.pdf]

Supplementary Materials: Assessing Community Readiness to Reduce Childhood Diarrheal Disease and Improve Food Security in Dioro, Mali

Erica C. Borresen, Cordelia Stone, Abdoulaye Boré, Alima Cissoko, Ababacar Maiga, Ousmane A. Koita and Elizabeth P. Ryan

Key Respondent Interview Introduction and Questions for Assessing Community Readiness to Reduce Childhood Diarrheal Disease in Dioro, Mali

1. Introduction

Please answer these questions pertaining only to Dioro in the Segou Region of southern-central Mali.

In addition, I would like to ask some questions regarding the local food system in Dioro, including rice production and milling.

For the following question, please answer keeping in mind your perspective of what Dioro residents believe and not what you personally believe.

2. Assessing Community Readiness to Reduce Childhood Diarrheal Disease Questionnaire

1. On a scale from 1–10, with 1 being "not a concern at all" and 10 being "a great concern"…?
(Scorer not: Community Climate)
…how much of a concern is childhood diarrheal diseases to residents of Dioro?

1	2	3	4	5	6	7	8	9	10
No concern									Great concern

Can you tell me why you think it's at that level?

Interviewer: Please ensure that the respondent answers the question in regards to community members not in regards to themselves or what they think it should be.

I'm now going to ask you about any current efforts in Dioro to reduce childhood diarrheal diseases. By efforts, I mean any programs, activities, or services occurring in Dioro that address reducing childhood diarrheal diseases, such as oral rehydration salts, hygiene practices (e.g., safe drinking water, hand washing), or feeding behaviors (e.g., breastfeeding).

2. Are there efforts in Dioro that are working to reduce childhood diarrheal diseases?

If yes, continue to question 3; if No, skip to Question 10.

3. Can you briefly tell me what each of these are and how long they've been going on?

Interviewer: Write down names of efforts so that you can refer to them in #4-5 below.

Effort 1:____
Effort 2:____
Effort 3:____
Effort 4:____

The next few questions are about awareness of these efforts. Awareness can range from having heard of a program to knowing how the program works and who it's targeted to.

4. For each of the efforts that you just mentioned, please tell me whether none, a few, some, or most community members have the following knowledge about that effort. If an effort is targeted to a specific

area in Dioro, please answer with respect to that area.

(Interviewer: Prompt them with the name of each effort, from what you wrote down before.)

Effort 1: ______________________________

	None	Few	Some	Most
Have heard of				
Purpose				
Who it's for				
How it works				

Effort 2: ______________________________

	None	Few	Some	Most
Have heard of				
Purpose				
Who it's for				
How it works				

Effort 3: ______________________________

	None	Few	Some	Most
Have heard of				
Purpose				
Who it's for				
How it works				

Effort 4: ______________________________

	None	Few	Some	Most
Have heard of				
Purpose				
Who it's for				
How it works				

5. Thinking back to your answers, why do you think members of your community have this amount of knowledge?

6. Are there misconceptions or incorrect information among community members about these current efforts? If yes: What are these?

7. How do community members in Dioro learn about these efforts?

8. Is there a need to expand education about these efforts?

9. What planning for additional efforts to address reducing childhood diarrheal diseases is occurring in Dioro?

Only ask #10 if the respondent answered "No" to #2 or was unsure.

10. Is anyone in Dioro trying to get something started to reduce childhood diarrheal diseases? Can you tell me about that?

LEADERSHIP QUESTIONS

For the next few questions, I am going to ask you how the leadership living in Dioro perceives the issue of reducing childhood diarrheal diseases. By leadership, we are referring to those who could affect the outcome of this issue and those who have influence in the Dioro community and/or who lead the community in helping it achieve its goals.

11. On a scale from 1–10, with 1 being "not a concern at all' and 10 being "a great concern"…
…how concerned is the leadership about childhood diarrheal diseases?

1	2	3	4	5	6	7	8	9	10
No concern									Great concern

Probe: Can you tell me why you gave that answer?

12. How are the leaders involved in efforts regarding reducing childhood diarrheal diseases? (Probe: Do they have a task force, do they provide resources, or do they serve on committees?)

13. I'm going to read a list of ways that community leaders might show their support or their lack of support for current, expanded, or new community efforts to address the issue of reducing childhood diarrheal diseases.

Can you please tell me whether none, a few, some, or most leaders would or do show their support in this way? Also, feel free to explain your responses as we move through the list.

How many community leaders…

- Speak out publicly in favor of efforts, for example at community gatherings or in the media? Prompt: none, a few, some, or most?
  None Few Some Most

- Play a key role as a leader or driving force in planning, developing, or implementing efforts?
  None Few Some Most

- Play a key role in ensuring the long-term viability of efforts?
  None Few Some Most

- Passively support efforts without being active in that support?
  None Few Some Most

- Actively oppose efforts, for example, by speaking out against them?
  None Few Some Most

- Passively oppose efforts?
  None Few Some Most

14. On a scale of 1 to 10, where 1 is 'not supportive' at all and 10 is 'very supportive', how supportive would leaders be of expanded or new efforts to reduce diarrheal diseases in Dioro?

1 2 3 4 5 6 7 8 9 10
Not a supportive at all Very supportive

Probe: How might they show this support (or lack of support)? For example, by passively supporting, by being involved in developing the efforts, by providing funding for the efforts, or by being a driving force or key player in achieving these expanded efforts?

15. Thinking back to your answers about leadership, on a scale from 1–10, with 1 being "not a priority at all" and 10 being "a very great priority"…
…how much of a priority is it to the leadership to address reducing childhood diarrheal diseases in the community?

1 2 3 4 5 6 7 8 9 10
Not a priority Great priority

Probe: Can you tell me why you gave that answer?

16. Who are the leaders specific to reducing childhood diarrheal diseases in your community? (no names are needed, just positions)

COMMUNITY CLIMATE

I'm now going to ask some questions about the attitudes and knowledge about Dioro residents toward the issue of reducing childhood diarrheal diseases.

17. Keeping in mind what residents of Dioro think and not what you think personally, on a scale from 1–10, with 1 being "not a priority at all" and 10 being "a very great priority"…

…how much of a priority is it to the residents of Dioro to address reducing childhood diarrheal diseases?

1 2 3 4 5 6 7 8 9 10

Not a priority Great priority

Probe: Can you tell me why you gave that answer?

18. I'm going to read a list of ways that community members might show their support or their lack of support for community efforts to reduce childhood diarrheal diseases?

Can you tell me whether none, a few, some, or most community members would or do show their support in this way? Also, feel free to explain your responses as we move through the list.

How many community members would or do…

- Passively support community efforts without being actively involved in the efforts?

Prompt: none, a few, some, or most community members

None Few Some Most

- Participate in developing, improving, or implementing efforts, for example by attending committee or group meetings that are working toward these efforts?

None Few Some Most

- Actively oppose community efforts, for example, by speaking out against them?

None Few Some Most

- Passively or silently oppose community efforts?

None Few Some Most

19. About how many community members would support expanding efforts in the community to address reducing childhood diarrheal diseases in Dioro? Would you say none, a few, some, many or most?

20. What are the primary obstacles to addressing reducing childhood diarrheal disease in Dioro?

21. Overall, thinking back on your answers, what would you say is the community's attitude about reducing childhood diarrheal diseases?

KNOWLEDGE ABOUT THE ISSUE

22. On a scale from 1 to 10, where 1 is "no knowledge" and 10 is "detailed knowledge"

…how much do residents of Dioro know about the importance of reducing childhood diarrheal diseases?

1 2 3 4 5 6 7 8 9 10

No knowledge Detailed knowledge

Why do you say it's a _____?

23. Would you say that community members know nothing, a little, some, or a lot about each of the following as they pertain to reducing childhood diarrheal diseases?

- How to reduce childhood diarrheal diseases?
  Nothing A little Some A lot

- How to find personnel/resources that can help reduce childhood diarrheal diseases?
  Nothing A little Some A lot

- Why reducing childhood diarrheal diseases is important?
  Nothing A little Some A lot

24. What types of information are available to residents of Dioro about reducing childhood diarrheal diseases/ (e.g., newspaper articles, brochures, posters, SMS)?

If they list information, ask: Do community members access and/or use this information?

25. Where would someone go first to get information on reducing childhood diarrheal diseases?
RESOURCES FOR REDUCING CHILDHOOD DIARRHEAL DISEASE

26. How are current efforts to reduce childhood diarrheal disease funded? If they know, ask: Is this funding likely to continue in the future?

29. I'm now going to read you a list of resources that could be used to address reducing childhood diarrheal diseases in your community. For each of these, please indicate whether there is none, a little, some, or a lot of that resource available in your community that could be used to address this issue?

- Community Health Workers or Doctors? Prompt: none, a little, some, or a lot of that resource is available?
  None Few Some A lot

- Financial donations from non-governmental organizations (NGOs) and/or international aid?
  None Few Some A lot

- Oral Rehydration Salts?
  None Few Some A lot

- Vaccinations to prevent diarrhea?
  None Few Some A lot

- Clean water?
  None Few Some A lot

- Safe, nutritious foods?
  None Few Some A lot

28. What is the level of expertise and training among those working on reducing childhood diarrheal diseases in Dioro? Please explain.

29. What is the most important thing that influences reducing childhood diarrheal diseases in your community? (e.g., hygiene, nutrition, medicine, *etc.*)

3. Assessing Community Readiness to Improve Food Security Questionnaire

1. Using a scale from 1–10, how much of a concern is food insecurity to Dioro with 1 being "not a concern at all" and 10 being "a very great concern"? Please explain (D)

DIMENSIONS A & B: COMMUNITY EFFORTS (programs and activities) and COMMUNITY KNOWLEDGE OF EFFORTS

2. Please describe the efforts (programs or activities) to improve food security and nutrition. (A)

(If Yes, continue to question 3; if No, skip to question 10)

3. How long have these efforts been going on in Dioro? (A)

4. Are there any segments of the community for which these efforts are or may appear inaccessible or unavailable? (For example, individuals of a certain age group, ethnicity, income level, geographic region) (A)

5. What are the strengths of these efforts? (A)

6. What are the weaknesses of these efforts? (A)

7. Using a scale from 1–10, how aware are people in this community of the efforts to reduce food insecurity (with 1 being no awareness to 10 being very aware)? Please explain. (B)

8. What do these individuals know about these efforts or activities?

9. Is there information available to the people living in Dioro about the efforts? Do community members take advantage of this information?

Respondent answered "No" to #2

10. Is anyone in Dioro trying to get something started to reduce food insecurity? If so, please explain.

DIMENSION C: LEADERSHIP

I'm going to ask you how the leadership in your community perceives this issue. By leadership, we are referring to those who could affect the outcome of this issue and those who have influence in the community and/or who lead the community in helping it achieve its goals in relation to the issue of food security.

11. Does leadership believe that food insecurity is an issue that needs addressing in Dioro? Please explain. If so, which leaders believe that it is?

12. How are the leaders involved in efforts to reduce food insecurity? For example, are leaders merely supportive or are they more actively involved, e.g., are they involved in a committee, do they speak out publicly, have they allocated resources to address the issue? (C)

13. Would the leadership support additional efforts? If so, how might they do that? (C)

DIMENSION D: COMMUNITY CLIMATE

14. Is reducing food security a priority to community members? Please explain.

15. Does the Dioro community support addressing reducing food insecurity locally? If yes, how might they show this support, e.g., passively or actively by being involved? (D)

DIMENSION E: KNOWLEDGE ABOUT THE ISSUE

16. How much do community members know about food insecurity in general? (e.g., the consequences of malnutrition in children during development) (E)

17. What type of information is available in your community about food insecurity (e.g., word of mouth, radio reports, newspaper articles, brochures, posters)? (E)

18. Are there local data available on how common food insecurity is in Dioro? (E) If so, how do people obtain this information?

DIMENSION F: RESOURCES FOR EFFORTS (time, money, people, space, *etc.*)

19. Who would an individual affected by food insecurity turn to first for help in this community? Why? (F)

20. What is the community's and/or local business' attitude about supporting efforts to reduce food insecurity? (F)

Additional questions:

21. Who are the leaders specific to improving food security in Dioro? (C)

22. What are the primary obstacles to addressing this issue in this community?

23. Describe the Dioro community in regards to food access and food affordability.

24. Describe typical family meals in this community. Do these meals vary between adults and children within the family? Male and females?

25. Is rice (full grain brown rice and/or white rice) a common food ingredient in families in this community?

26. Who is the primary person in charge of purchasing (or growing) food in the family?

27. What is the most important component of food products in this community? (e.g., taste, nutrition, price, access, culturally-important, *etc.*)

28. Would this community be interested in modifying ingredients used in traditional foods (like porridge)?

Questions related to malnutrition

29. How do you define malnutrition?

30. How do treat the malnourished children?

© 2016 by the authors; licensee MDPI, Basel, Switzerland. This article is an open access article distributed under the terms and conditions of the Creative Commons by Attribution (CC-BY) license (http://creativecommons.org/licenses/by/4.0/).
